# Intestinal injury and vasculitis biomarkers in cats with feline enteric coronavirus and effusive feline infectious peritonitis

**DOI:** 10.1002/vms3.1299

**Published:** 2023-10-24

**Authors:** Erdem Gülersoy, Mahmut Ok, Kamil Üney, Murat Kaan Durgut, Tuğba Melike Parlak, Yusuf Emre Ekici

**Affiliations:** ^1^ Department of Internal Medicine Veterinary Faculty Harran University Şanlıurfa Turkey; ^2^ Department of Internal Medicine Veterinary Faculty Selçuk University Konya Turkey; ^3^ Department of Pharmacology and Toxicology Veterinary Faculty Selçuk University Konya Turkey

**Keywords:** ANCA, feline coronavirus, FIP, IAP, I‐FABP, TFF‐3

## Abstract

**Objective:**

To investigate intestinal injury, repair and vasculitis biomarkers that may illuminate the progression and/or pathogenesis of feline infectious peritonitis (FIP) or feline enteric coronavirus (FECV) infection.

**Materials and methods:**

A total of 40 cats with effusive FIP (30 with abdominal effusion, AE group; 10 with thoracic effusion, TE group) and 10 asymptomatic but FECV positive cats (FECV group), all were confirmed by reverse transcription polymerase chain reaction either in faeces or effusion samples. Physical examinations and effusion tests were performed. Trefoil factor‐3 (TFF‐3), intestinal alkaline phosphatase (IAP), intestinal fatty acid binding protein (I‐FABP), myeloperoxidase‐anti‐neutrophilic cytoplasmic antibody (MPO‐ANCA) and proteinase 3‐ANCA (PR3‐ANCA) concentrations were measured both in serum and effusion samples.

**Results:**

Rectal temperature and respiratory rate were highest in the TE group (*p* < 0.000). Effusion white blood cell count was higher in the AE group than TE group (*p* < 0.042). Serum TFF‐3, IAP and I‐FABP concentrations were higher in cats with effusive FIP than the cats with FECV (*p* < 0.05). Compared with the AE group, TE group had lower effusion MPO‐ANCA (*p* < 0.036), higher IAP (*p* < 0.050) and higher TFF‐3 (*p* < 0.016) concentrations.

**Clinical significance:**

Markers of intestinal and epithelial surface injury were higher in cats with effusive FIP than those with FECV. Compared to cats with abdominal effusions, markers of apoptosis inhibition and immunostimulation to the injured epithelium were more potent in cats with thoracic effusion, suggesting the possibility of a poorer prognosis or more advanced disease in these patients.

## Graphical abstract

Feline enteric coronavirus (FECV) and feline infectious peritonitis (FIP) exhibit functional differences, as FIP virus in cats is believed to be a mutated FECV, and FECV causes clinical symptoms associated with gastroenteritis and FIP virus causes systemic disease, certain biomarkers may be of interest in terms of diagnosis and/or prognosis and development of FIP. As a result, it was thought that suppressing the expression of proteins such as intestinal alkaline phosphatase (IAP) and trefoil factor 3 (TFF‐3), which are involved in immunostimulation, and stimulating the expression of proteins such as intestinal fatty acid binding protein (I‐FABP) may shed light on preventing the progression of FECV and predicting the prognosis in cats with effusive FIP.

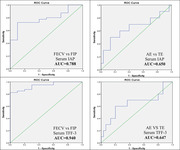

## 1 INTRODUCTION

Feline enteric coronavirus (FECV), defined as the ubiquitous enteric biotype, and feline infectious peritonitis virus (FIPV), the virulent biotype that causes feline infectious peritonitis (FIP), are two pathotypes of feline coronavirus (FCoV) (Kipar & Meli, 2014; Pedersen, 2009). FECV and FIP exhibit functional differences; FECV replicates mainly in the intestinal epithelium, whereas FIPV replicates efficiently in monocytes thus promoting systemic disease. FECV infection is transmitted via the faeco‐oral route. It is rarely fatal when in its native biotype (Rush et al., 1990) but may cause mild gastroenteritis. In contrast, FIPV is rarely transmitted from cat to cat and develops “de novo” as a result of spontaneous mutation within an FECV‐infected host (Hartmann, 2005; Pedersen et al., 2015). Inflammation plays a significant role in the pathogenesis of FIP (Hartmann, 2005). The major lesions include severe systemic inflammatory damage of serosal membranes (Kipar & Meli, 2014), widespread pyogranulomatous lesions (Felten & Hartmann, 2019), vasculitis (Pedersen, 2014) and occasionally mild gastroenteritis (Hartmann, 2005; Pedersen et al., 2015). Highly proteinaceous body cavity effusions are a common sequela.

Intestinal fatty acid binding protein (I‐FABP), which is localized in enterocytes, enters the blood circulation as a result of cell injury/destruction (Abdel‐Haie et al., 2017) and can be used in the evaluation of intestinal injury (Gülersoy et al., 2020; Ok et al., 2020; Yıldız et al., 2018; Yıldız & Ok, 2022). Trefoil factors (TFF‐1, 2 and 3) are peptides that play a role in the protection and repair of epithelial surfaces, including the gastrointestinal tract (Aamann et al., 2017). They serve to increase mucus viscosity, facilitate cell migration, inhibit apoptosis and affect the immune response (Hoffmann, 2021). Likewise, intestinal alkaline phosphatase (IAP) is expressed in the intestinal epithelium to alleviate intestinal and systemic inflammation (Fawley & Gourlay, 2016) and is actively found in serum (Lallès, 2014). The predominant cells found in granulomatosis with polyangiitis, granulomatous lesions and small vessel vasculitis are anti‐neutrophilic cytoplasmic antibody (ANCA)‐activated polymorphonuclear cells (Jennette et al., 2013). It was reported that proteinase 3‐ANCA (PR3‐ANCA) and myeloperoxidase‐ANCA (MPO‐ANCA) are not only important for the characterization of vasculitis but also have diagnostic and prognostic utility in diseases that cause systemic granulomatous vasculitis (Kipar et al., 2005; Schönermarck et al., 2014).

Feline gastroenteritis is a common clinical problem (Hall & German, 2005). As FECV is a common cause of feline enteritis and FIPV is believed to be mutated from FECV, such biomarkers may be of diagnostic and/or prognostic interest. Therefore, the aim of this study was to investigate the concentrations of intestinal injury (I‐FABP), repair (TFF‐3, IAP) and granulomatous vasculitis (PR3‐ANCA, MPO‐ANCA) biomarkers to illuminate the progression and/or pathogenesis of FIP and FECV infection.

## 2 MATERIALS AND METHODS

### 2.1 Ethical considerations

This study was approved by the Ethics Committee of Selçuk University, Faculty of Veterinary Medicine, Experimental Animal Production and Research Center (SUBAPK) with a 2020/43 committee decision.

### 2.2 Animal material

The study sample comprised 50 cats naturally infected with FCoV in total; 40 cats with effusive FIP (FIP group) and 10 asymptomatic but FECV positive cats (FECV group). All cats included in the study were recruited from the cats admitted to the animal hospital either for routine check‐up and diagnostic and/or treatment purposes between 2020 and 2021 subject to owner consent.

### 2.3 Inclusion/exclusion criteria

All 50 cats were evaluated by anamnesis, physical examination, complete blood count, urinalysis, microscopic evaluation of faeces, serum biochemistry, infectious disease screening, radiographic and ultrasonographic examinations in order to rule out any co‐morbidities as the asymptomatic cats had mild diarrhoea in their medical history. Feline parvovirus (FPV), feline leukaemia virus (FeLV) and feline immunodeficiency virus (FIV) testing were done using rapid diagnostic test kits (IDEXX SNAP Feline Parvo and FIV/FeLV Combo Test, IDEXX Laboratories) measuring antigen for FPV, p27 antigen for FeLV and antibodies for FIV. The results of these diagnostic tests were used only as a tool for inclusion/exclusion and were not further evaluated in the present study. A diagnosis of effusive FIP was made based on the presence of a high FCoV viral load in the effusion and the presence of compatible clinical signs (Felten & Hartmann, 2019). FECV infection was assumed based on a high FCoV viral load in the faeces, a lack of lymphopenia (<1.5 cells/μL) and absence of clinical signs (Tobler et al., 1993; Dye et al., 2008). Exclusion criteria included comorbid disease (such as pancreatitis, lymphocytic cholangitis, toxoplasmosis, retroviral infection, sepsis, septic peritonitis, endoparasites, having diarrhoea and abdominal/thoracic carcinoma), neurological signs (such as seizures, abnormal mental status, abnormal behaviour, cranial nerve deficits, central vestibular signs, ataxia, tetraparesis and hyperesthesia) and ocular signs (such as unilateral or bilateral anterior uveitis manifested by change in iris colour, flocculant debris in the anterior chamber, keratic precipitates, anisocoria and retinal changes including blood vessel cuffing and sheathing, retinal detachment and retinitis) that might suggest the dry form of FIP and being under treatment with drugs such as antivirals, antibiotics, antihistamines, corticosteroids, non‐specific immunostimulants or nonsteroidal anti‐inflammatory drugs.

### 2.4 Physical examinations

A comprehensive physical examination, including the measurement of rectal temperature, gingival capillary refill time (CRT), heart and respiratory rate, was performed in each cat. Radiographic examinations of the thorax and abdomen were performed using a Polydoros generator (Siemens) with settings of 40 kv, 12.5 mAs and 18.9 ms, a Multix table (no grid) (Siemens), an imaging plate of 35.4 × 43.0 cm^2^, DI‐AT film (dry, 35 × 43 cm^2^) (Fuji) and a digital FCR AC‐3CS reader (Fuji). The cats were gently restrained on the table with the forelimbs extended cranially and the hindlimbs caudally with minimal patient stress. The exposure was performed with full inspiration whenever possible. Ultrasonographic (Mindray z60) examinations were performed through the subxiphoid, splenorenal, hepatorenal and systolic window for the abdomen; bilateral dynamically focused pericardial and diaphragmatic regions were employed for the thorax either using a 5.0 or 7.5 MHz microconvex probe. Radiographic imaging and ultrasonographic imaging were performed in all cats to confirm the presence and localization of effusion and were not further evaluated in the present study.

### 2.5 Sampling and storage

Blood and effusion samples were taken unsedated by a single clinician with minimal restraint to avoid stress. Blood samples (3–5 mL) were taken from the vena saphenous or vena cephalica via venipuncture. Abdominal or thoracic effusion samples (2–3 mL) were obtained percutaneously under ultrasound guidance following standard sterility protocols and using 22‐gauge over‐the‐needle catheter, at the seventh–nine intercostal space to avoid the heart or liver. Blood samples were transferred into tubes without anticoagulant and were centrifuged at 5000 rpm for 5 min following which the serum supernatant was transferred to Eppendorf tubes. Effusion samples were collected into sterile tubes with ethylenediaminetetraacetic acid to prevent clot formation that may alter cell count. Serum and effusion samples were stored at −20°C up to 30 days following sampling.

### 2.6 Viral RNA extraction using RT‐PCR

All reverse transcription polymerase chain reaction (RT‐PCR) analyses were carried out in the central laboratory of Harran University (Supporting Information). For the detection of FCoV from effusion samples, the procedure was performed as described previously (Dye et al., 2008; Toussaint et al., 2007). Briefly, viral RNA was extracted from cell‐free abdominal/thoracic effusions using a QIAamp Viral RNA Kit (Qiagen). In order to inactivate RNases and isolate the intact viral RNA, 140 μL aliquots of the samples were lysed under high denaturing conditions. The silica membrane of the QIAamp Mini spin column grants optimal binding under modified buffering conditions. The RNA was eluted with 60 μL RNase‐free buffer, washed twice with wash buffers and stored at −80°C. The one‐step RT‐PCR was carried out using the QuantiTect Probe RT‐PCR kit (Qiagen). All primers had a concentration of 0.8 μM, and 5′FAM/3′BHQ‐1‐labelled TaqMan probes (Applied Biosystems) had a concentration of 0.3 μM. For the detection of FCoV from cats in the FECV group, the same RNA extraction procedure was followed using the QIAamp viral RNA mini kit as described previously (Dye et al., 2008). Viral RNA was extracted from 200 mL of a 10% faecal suspension in phosphate‐buffered saline using the QIAamp mini kit according to the manufacturer's instructions. Faecal samples were suspended in ASL buffer (Stool Lysis Buffer, Qiagen) to remove inhibitory substances. After the centrifugation (20 s at 16,000*g*), substances were adsorbed. Following proteinase K (CAS 39450‐01‐6, BRENDA) treatment, the samples were bound to a silica‐gel‐based capture membrane, washed, eluted in a low‐salt buffer and stored at −80°C. A 20 mL aliquot of extraction matrix was added to 10% faecal suspension, vortexed and suspended at room temperature for 15 min. The RNA‐containing supernatant obtained following the boom method was evaluated with the Primer3 software package (University of Tartu, Estonia, access number DQ010921). Superscript II RNAse H^−^ reverse transcriptase (Invitrogen) was used to reverse transcribe viral RNA. Real‐time PCR reactions were done in duplicate using HotStarTaq master mix (Qiagen) according to the manufacturer's instructions. Following reverse transcription the cDNA was diluted 1:1000 with AE buffer (10 mM Tris–Cl; 0.5 mM EDTA; pH 9.0) and 5 mL was used as template in the real‐time PCR assay.

### 2.7 Effusion tests

The gross appearance of the effusion (such as viscid, yellow‐tinged, cloudy appearance) was noted. Effusion specific gravity (Sg), pH, total protein and white blood cell (WBC) were evaluated with both dipstick (URIT‐31) and refractometer (Kruuse) examinations. Rivalta testing was performed on all the effusion samples.

### 2.8 Biomarker measurements of serum and effusion samples

Concentrations of TFF‐3, IAP, I‐FABP and MPO‐ and PR3‐ANCA were measured both in serum and effusion samples using commercial feline specific sandwich assay type qualitative enzyme‐linked immunosorbent assay (Bioassay Technology Laboratory; DRG International Inc, respectively) test kits. The reported intra‐ and inter‐assay coefficients of variation (CV) for TFF‐3 were 8% and ≤10%, respectively; and the minimum detectable concentration (MDC) was 0.093 ng/mL, and detection range was 0.2–70 ng/mL. The intra‐ and inter‐assay CV reported for IAP were 8% and ≤10%, respectively; and the MDC was 0.012 ng/mL, and detection range was 0.05–15 ng/mL. The intra‐ and inter‐assay CV reported for I‐FABP were 8% and ≤10%, respectively, and the MDC was 0.52 ng/mL, with a detection range of 1–400 ng/mL. The intra‐ and inter‐assay CV reported for MPO‐ANCA were ≤6.4% and ≤6.3%, respectively; and the MDC was 0.5 U/m, and the detection range was 0–100 U/mL. Reported intra‐ and inter‐assay CV for PR3‐ANCA were ≤4.7% and ≤8.8%, respectively; and the MDC was 0.5 U/L, and detection range was 0–100 U/mL.

### 2.9 Statistical analysis

All data were evaluated using SPSS 25.00 (SPSS for Windows) statistical software. One sample Kolmogorov–Smirnov test was used to determine whether the data were parametric. Parametric data were presented as mean ± SD, and non‐parametric data were presented as median (min, max) and were inspected with Mann–Whitney *U* and Kruskal–Wallis tests, respectively. Statistical significance between means of the groups was investigated using one‐way ANOVA with post hoc Tukey. Receiver operating characteristic (ROC) curve analysis was used to investigate the diagnostic and/or prognostic efficacy of the aforementioned biomarkers. Diagnostic performances of biomarkers were evaluated with parameters including area under the curve (AUC, >0.600), standard deviation (Std. error), diagnostic sensitivity and specificity (>70%), accuracy (%), positive predictive value (%), negative predictive value (%) and power analyses (Op, %) for serum samples; AUC, Std. error, diagnostic sensitivity and specificity and Op values for effusion samples. It was accepted that AUC of 0.5 suggests no discrimination (i.e. ability to diagnose patients with and without the disease or condition based on the test), 0.6–0.8 was considered acceptable, 0.8–0.9 excellent and >0.9 outstanding (Hosmer & Lemeshow, 2000). Statistical significance was accepted as *p* < 0.05 for all data.

## 3 RESULTS

### 3.1 Clinical findings

All cats were client‐owned indoor cats living in single cat household and fed on commercial dry cat food. The 40 cats with FIP included 26 males and 24 females between 1 and 2 years of age and comprised the following breeds: 31 mixed breed, 4, Scottish, 2 British Longhair, 2 Persian and 1 Bombay cat. The 10 asymptomatic cats included 7 males and 3 females between 1 and 2 years of age and comprised the following breeds: 8 mixed breed, 1 Scottish Fold and 1 Bombay cat. Most (44/50) cats were unvaccinated, and the remaining 6 cats were vaccinated only once with Felocell CVR 3 (Zoetis) containing attenuated strains of feline rhinotracheitis virus, calicivirus and panleukopenia virus (Johnson Snow Leopard strain). Screening tests for FPV, FeLV and FIV were negative in all cats. Of the cats with effusive FIP, abdominal distension, laboured breathing and solitary lymph node enlargement were common. The average duration of clinical signs was longer for cats with abdominal effusion (11.5 days, range 5–22) compared with those that had thoracic effusion (8.5 days, range 5–22) (*p* < 0.000). Compared with the AE group, cats in the TE group had higher rectal temperature (*p* < 0.000), respiratory rate, pulse rate (*p* < 0.000) and CRT (*p* < 0.019). Physical examination findings are presented in Table 1.

**TABLE 1:** Physical examination findings.

		FIP group				
Parameters	FECV group *n* = 10 mean ± SD	AE group *n* = 30 mean ± SD	TE group *n* = 10 mean ± SD	F	*p* Value	Post hoc Tukey's test
RBT (°C)	38.54 ± 0.29^c^	39.04 ± 0.36^b^	39.58 ± 0.30^a^	7.563	0.000	*a* > *b* > *c*
RR (breaths/min)	38.8 ± 5.75^c^	68.07 ± 13.24^b^	93.60 ± 11.26^a^	7.422	0.000	*a* > *b* > *c*
PR (beats/min)	131.6 ± 10.31^b^	126.47 ± 12.52^b^	159.60 ± 7.64^a^	7.610	0.000	*a* > *b*
CRT (s)	2.40 ± 0.51^b^	2.87 ± 0.77^a,b^	3.30 ± 0.48^a^	4.165	0.019	*a* > *b*

*Note*: To explore differences between multiple group means, one‐way ANOVA with post hoc Tukey test was utilized. a, b, c: Compact letters are used to show the significant differences between the groups.

Abbreviations: CRT, capillary refill time; PR, pulse rate; RBT, rectal body temperature; RR, respiratory rate.

### 3.2 Effusion tests and cytological examination of effusion samples

Effusions were poorly cellular (<5 × 10^9^ cells/L) and were typically pyogranulomatous in nature with a few non‐degenerate neutrophils and very few lymphocytes. Neither free or phagocytized bacteria nor nuclear degenerations were observed. Effusion WBC was higher in the AE group than TE group (*p* < 0.042). No other statistical differences were observed. Effusion test analyses findings are presented in Table 2.

### 3.3 Serum biomarker analysis findings

IAP, TFF‐3 and I‐FABP concentrations of the FIP group were higher than those of the FECV group (*p* < 0.010). The serum biomarker analysis findings are presented in Table 3.

### 3.4 Effusion biomarker analysis findings

Compared with the AE group, the TE group had lower MPO‐ANCA (*p* < 0.036), higher IAP (*p* < 0.050) and higher TFF‐3 (*p* < 0.016) concentrations. The effusion biomarker analysis findings of the FIP group are presented in Table 4.

### 3.5 ROC analysis findings

**FIGURE 1:**
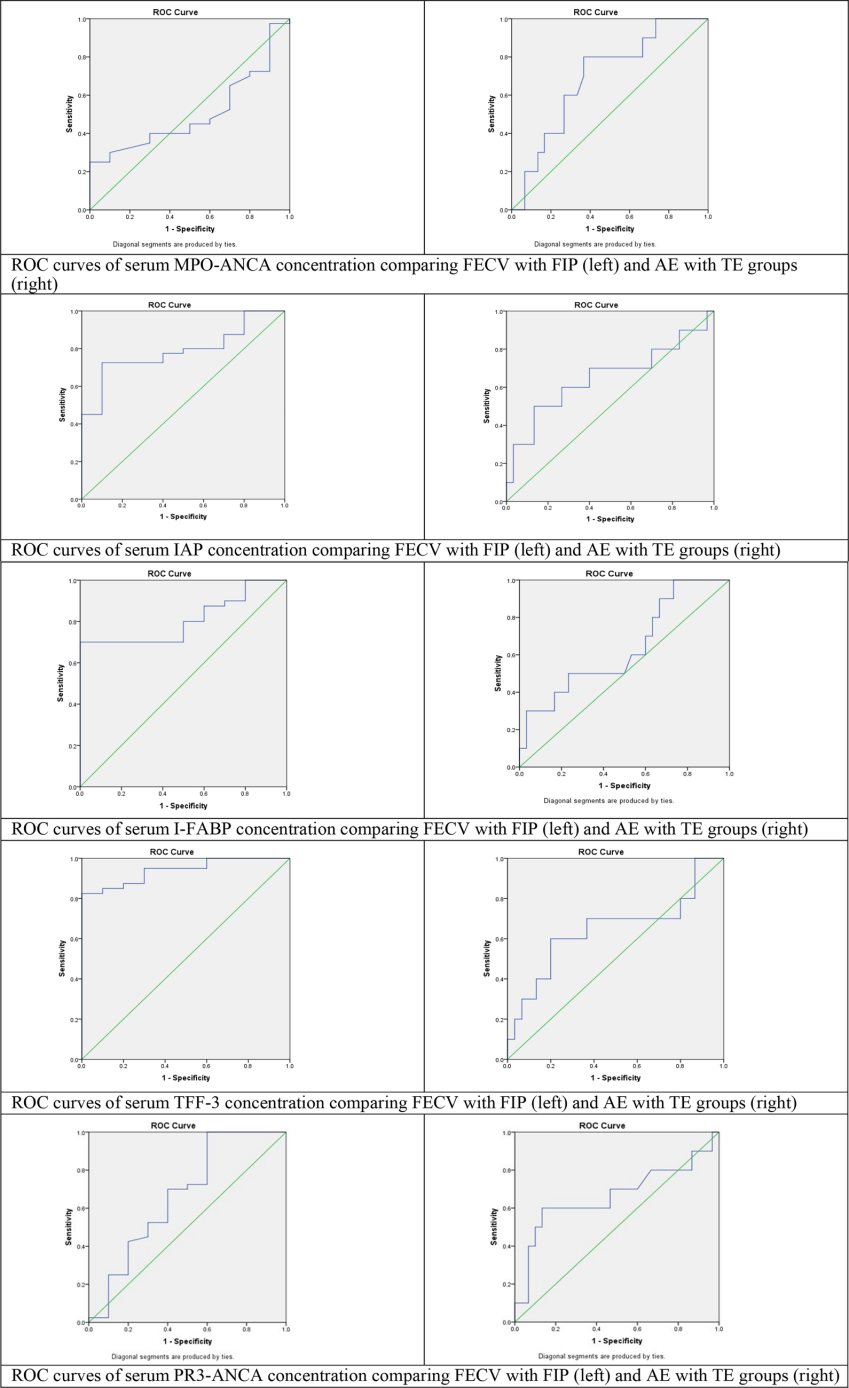
Comparative receiver operating characteristic (ROC) curves of serum biomarkers (diagonal segments are produced by ties).

In the FIP and FECV group comparisons, serum IAP, I‐FABP and PR3‐ANCA had acceptable, and TFF‐3 had outstanding diagnostic performance. MPO‐ANCA had no discriminative diagnostic utility. All serum analytes exhibited acceptable diagnostic performance for distinguishing the AE and TE groups. ROC analysis results of serum biomarkers between the FECV and FIP groups are shown in Table 5, and results between AE and TE groups are shown in Table 6. The comparative ROC curves of the FIP and FECV and of the AE and TE groups are presented in Figure 1.

## 4 DISCUSSION

The clinical presentation of FIP is complex and diverse, and this diversity may be a result of variations in the virus and in the nature of the individual host's immune response (Yin et al., 2021), particularly in relation to T‐cell depletion and viral load (Kipar & Meli, 2014). In this study, asymptomatic cats, cats with TE and those with AE could be differentiated based on their vital parameters (Table 1). Intuitively, respiratory rate was highest in cats with TE and, interestingly, this group also had the highest heart rates, CRT and rectal temperature. The severity of clinical findings of the TE group is likely to be related to gas exchange impairment due to the alteration of the ventilation‐perfusion mechanism and consequently hypoperfusion due to thoracic effusion (Thomas et al., 2015). Moreover, earlier admission to the hospital may be a factor, as laboured breathing can be noticed more easily by owners. Concurrently, the higher effusion WBC, IAP and TFF‐3 in this group suggest enhanced epithelial inflammation in cats with thoracic effusion which may have a direct effect on vital signs.

**TABLE 2:** Effusion tests results.

	FIP group		
Parameters	AE group *n* = 30 median (min, max)	TE group *n* = 10 median (min, max)	*p* Value
Eff. Sg	1.030 (1.020, 1.030)	1.030 (1.020, 1.30)	0.829
Eff. pH	7 (7, 7)	7 (6.50, 7)	0.071
Eff. total protein	3 (2, 3)	3 (1, 6)	0.728
Eff. WBC	3 (2, 3)	2 (0, 3)	0.042

Abbreviations: Eff., effusion; pH: power of hydrogen; Sg, specific gravity; T. Pro, total protein; WBC, leukocyte.

**TABLE 3:** Serum biomarker analysis results.

		FIP group				
Parameters	FECV group *n* = 10 mean ± SD	AE group *n* = 30 mean ± SD	TE group *n* = 10 mean ± SD	*F*	*p* Value	Post hoc Tukey's test
MPO‐ANCA (U/mL)	0.75 ± 0.39	1.33 ± 2.58	1.33 ± 1.01	0.303	0.740	–
PR3‐ANCA (U/mL)	1.55 ± 0.95	1.85 ± 0.62	2.34 ± 0.90	2.831	0.069	–
IAP (ng/mL)	1.72 ± 0.48^b^	2.51 ± 1.07^a,b^	3.45 ± 1.86^a^	5.280	0.009	*a* > *b*
I‐FABP (ng/mL)	53.93 ± 23.73^b^	91.38 ± 50.39^a,b^	133.18 ± 86.16^a^	5.080	0.010	*a* > *b*
TFF‐3 (ng/mL)	4.88 ± 1.66^b^	11.42 ± 6.11^a^	15.84 ± 9.17^a^	7.733	0.001	*a* > *b*

*Note*: To explore differences between multiple group means, one‐way ANOVA with post hoc Tukey test was utilized. a, b, c: Compact letters are used to show the significant differences between the groups.

Abbreviations: IAP, intestinal alkaline phosphatase; I‐FABP, intestinal fatty acid binding protein; MPO‐ANCA, myeloperoxidase‐anti neutrophil cytoplasmic antibody; PR3‐ANCA, proteinase 3 anti‐neutrophil cytoplasmic antibody; TFF‐3, trefoil factor 3.

**TABLE 4:** Effusion biomarker analysis results.

	FIP group		
Parameters	AE group *n* = 30 mean ± SD	TE group *n* = 10 mean ± SD	*p* Value
MPO‐ANCA (U/mL)	1.15 ± 1.91	0.37 ± 0.25	0.036
PR3‐ANCA (U/mL)	1.69 ± 0.43	2.17 ± 1.03	0.178
IAP (ng/mL)	2.39 ± 0.36	2.72 ± 0.43	0.050
I‐FABP (ng/mL)	84.03 ± 25.93	99.34 ± 30.86	0.181
TFF‐3 (ng/mL)	10.31 ± 1.98	13 ± 2.80	0.016

Abbreviations: IAP, intestinal alkaline phosphatase; I‐FABP, intestinal fatty acid binding protein; MPO‐ANCA, myeloperoxidase‐anti neutrophil cytoplasmic antibody; PR3‐ANCA, proteinase 3 anti‐neutrophil cytoplasmic antibody; TFF‐3, trefoil factor 3.

**TABLE 5:** Receiver operating characteristic (ROC) analysis results of serum biomarkers between feline enteric coronavirus (FECV) and feline infectious peritonitis (FIP) groups.

				Asymp. 95% CI								
Parameters	AUC	Std. Error	*p* Value	Lower bound	Upper bound	Cut‐off	Sensitivity (%)	Specificity (%)	Acc (%)	PPV (%)	NPV (%)	Op (%)
MPO‐ANCA	0.503	0.090	0.981	0.326	0.679	0.63	47.5	40	46	76	16	65.9
PR3‐ANCA	0.666	0.111	0.107	0.449	0.883	1.59	70	60	68	87.5	33.3	63.4
I‐FABP	0.808	0.062	0.003	0.686	0.929	76.006	70	100	76	100	45.4	79.5
TFF‐3	0.940	0.032	0.000	0.877	1.000	7.24	82.5	100	86	100	58.8	93.6
IAP	0.788	0.068	0.005	0.655	0.920	1.95	72.5	90	76	96.6	45	83

Abbreviations: Acc, accuracy; AUC, area under curve; CI, confidence interval; IAP, intestinal alkaline phosphatase; I‐FABP, intestinal fatty acid binding protein; MPO‐ANCA, myeloperoxidase‐anti neutrophil cytoplasmic antibody; NPV, negative predictive value; Op, observed power; PPV, positive predictive value; PR3‐ANCA, proteinase 3 anti‐neutrophil cytoplasmic antibody; Std. error, standard error; TFF‐3, trefoil factor 3.

The intestinal lesions caused by FCoV typically comprise patchy mucosal changes and villous atrophy predominantly located in the ileum and distal duodenum. Focal fusion of adjacent villi is also reported (Kipar et al., 1998). In critically ill patients, the intestine is a vulnerable organ, and the status of the gastrointestinal tract is important in terms of clinical outcome (Li et al., 2017). In the presence of intestinal injury, intestinal biomarkers are released into the bloodstream. Of these, IAP increases in hunger, prematurity and inflammatory/infectious conditions (Fawley & Gourlay, 2016). It has anti‐inflammatory effects and may prevent or treat a large number of intestinal and extra‐intestinal diseases (Singh & Lin, 2021). TFF‐3 was reported to be increased in dogs with parvoviral enteritis (Gülersoy et al., 2020), and in calves with atresia coli (Yıldız et al., 2018) and with diarrhoea (Ok et al., 2020). Moreover, TFF‐3 and IAP concentrations may increase in the presence of hypoxia, hypoperfusion, mucosal damage and during active intestinal mucosal defence (Yıldız et al., 2018). Moreover, it was reported that TFFs modulate immune reactions by triggering inflammation (Yang et al., 2022). I‐FABP, which is primarily limited to the mature enterocytes of the small intestine, was reported to be increased in cases of acute ischemic bowel injury (Gollin et al., 1993), necrotizing enterocolitis (Abdel‐Haie et al., 2017), atresia coli (Yıldız et al., 2018), enteritis (Ok et al., 2020) and parvoviral enteritis (Gülersoy et al., 2020).

In the present study, the FIP group had higher serum I‐FABP, IAP and TFF‐3 concentrations than the FECV group (*p* < 0.010, *p* < 0.009, *p* < 0.001, respectively). This indicates that the extension of the peritoneal inflammation involves the gastrointestinal tract (Sherding, 2006). Moreover, these findings suggest that, along with the development of extra‐intestinal pathology, intestinal and epithelial surface injuries are more severe in cats with effusive FIP than in those with FECV. In addition, effusion IAP and TFF‐3 concentrations were higher in the cats with thoracic effusion than those with abdominal effusion (*p* < 0.05 and *p* < 0.016, respectively). As previously proposed (Hartmann, 2005; Kipar & Meli, 2014), this suggests that responses such as apoptosis inhibition and immunostimulation to the injured epithelium were more potent in cats with thoracic effusion. Moreover, ROC analysis revealed that serum TFF‐3 had outstanding, I‐FABP had excellent, and IAP had acceptable to distinguish the AE and TE groups based on the AUC values (Table 6). These findings may also be a result of more severe inflammatory activity and increased acute phase response (Tamura et al., 1988) considering the higher effusion WBC in the cats with thoracic effusion. Higher serum and effusion TFF‐3 and IAP concentrations in the cats with thoracic effusion may also be associated with hypoxia due to the presence of effusion (Furuta et al., 2001). Highly virulent FIPV isolates have the ability to replicate efficiently in macrophages (Rottier et al., 2005). In granulomatous diseases of the pleura, the pleural fluid consists primarily of mononuclear cells, which are mostly macrophage‐like (Antony, 2003). Moreover, following inflammatory challenge, integrins such as very late antigen‐4 and ‐5 enhance the adhesion of peritoneal macrophages to the mesothelial wall of peritoneum (Bellingan et al., 2002). In accordance with this information, one can speculate that more pleural macrophages differentiating from peripheral blood monocytes migrate through the lining of the pleural mesothelium than those of peritoneal macrophages to peritoneal mesothelium (Kaczmarek & Sikora, 2012). Although this finding can be supported by the presence of higher thoracic effusion WBC level compared to its counterpart in the abdominal effusion, further studies including the investigation of cytokines and chemokines are recommended. It is well known that bacterial translocation is an important contributor to systemic inflammation in cases of intestinal injury, and without repair of the intestinal barrier, a continuous inflammatory stimulus is capable of triggering an autoimmune process (Rizzetto et al., 2018). As FIP is an immune complex disease (Addie et al., 2003; Berry, 2001), one can speculate that suppressing the expression of proteins such as IAP and TFF‐3 and stimulating the expression of proteins such as I‐FABP may affect disease progression.

**TABLE 6:** Receiver operating characteristic (ROC) analysis results of serum biomarkers between AE and TE groups.

Parameters	AUC	Std. Error	*p* Value	Asymp. 95% CI		Cut‐off	Sensitivity (%)	Specificity (%)	Op (%)
				Lower bound	Upper bound				
MPO‐ANCA	0.692	0.091	0.072	0.514	0.869	1.009	60	73.3	92.3
PR3‐ANCA	0.663	0.117	0.126	0.434	0.892	1.84	70	53.3	60.8
I‐FABP	0.638	0.103	0.195	0.436	0.840	111.94	50	76.6	86
TFF‐3	0.647	0.115	0.169	0.422	0.871	13.64	60	80	60.4
IAP	0.650	0.115	0.160	0.424	0.876	2.32	70	60	83

Abbreviations: AUC, area under curve; CI, confidence interval; IAP, intestinal alkaline phosphatase; I‐FABP, intestinal fatty acid binding protein; MPO‐ANCA, myeloperoxidase‐anti neutrophil cytoplasmic antibody; Op, observed power; PR3‐ANCA, proteinase 3 anti‐neutrophil cytoplasmic antibody; Std. error, standard error; TFF‐3, trefoil factor 3.

The most prominent features of FIPV infection are phlebitis and periphlebitis, ranging from granulomatous to necrotic in character (Kennedy, 2020; Quintana et al., 2014). In areas with vasculitis, the predominant cells are infected monocytes and lesser numbers of neutrophils and lymphocytes. Interestingly, the inflammatory process in FIP is restricted to small and medium veins (Kennedy, 2020). ANCAs are produced against antigens in the cytoplasm of neutrophil granulocytes and monocytes. ANCA‐associated vasculitis defines granulomatous vasculitis, and neutrophils and their products play a role in tissue damage and in granulomatous inflammation as well as initiation of autoimmune response (Schönermarck et al., 2014). PR3‐ANCA is associated with the granulomatous inflammation of the respiratory system and organs other than the kidney, whereas MPO‐ANCA is more associated with abdominal organs especially the kidney (Quintana et al., 2014). In the present study, no statistical differences were observed in terms of serum MPO‐ANCA and PR3‐ANCA concentrations between the FECV and FIP groups. Moreover, ROC analysis revealed indiscriminative diagnostic performance for serum MPO‐ANCA. This finding can be explained by the fact that the inflammatory infiltrate of FIP vasculitis has low neutrophil counts due to the absence of vascular cell adhesion protein 1 in neutrophils (Kipar et al., 2005). On the contrary, among all the groups, ROC analysis of serum PR3‐ANCA concentration revealed acceptable diagnostic performance. Considering the ROC analysis results of serum MPO‐ANCA, higher diagnostic sensitivity and specificity of serum PR3‐ANCA may be due to the stronger stimulation of the infectious antigen by proteinase‐3‐related autoimmunity. The fact that respiratory symptoms may also be seen in addition to mild vomiting and diarrhoea due to the replication of FCoV in enterocytes may also be related to this finding (Addie & Jarrett, 1990). Besides, this finding may be related to neutrophil extracellular traps and mechanisms that play a further role in the development of autoimmunity in cases of acute viral infection such as coronavirus disease 2019 (Kant et al., 2021), which has been reported to have similar pathogenic and immunopathogenic events as in FIP (Paltrinieri et al., 2021). In addition, as a result of ROC analysis of effusion samples between the AE and TE groups, acceptable diagnostic performances were determined only for MPO‐ANCA concentration. This finding was interpreted as an indicator of a possible presence of more severe abdominal organ/tissue damage including pancreas, gastrointestinal and hepatobiliary system as a result of the extension of the peritoneal inflammation in cases of FIP with abdominal effusion (Sherding, 2006).

MPO‐ and PR3‐ANCA are highly specific for polyangiitis which is characterized by the inflammation of small and medium veins that results in damage to various organ systems of the body, most often the respiratory tract and kidneys (Monach, 2014). Although FIP and polyangiitis cause similar clinical outcomes, the diagnostic and/or prognostic efficacies of serum MPO‐ and PR3‐ANCAs were found to be low, presumably due to their different pathogenesis as the predominant cells are monocytes and only a small number of neutrophils have been reported in FIP vasculitis (Rush et al., 1990). This study has some limitations, such as the limited number of animals, which might have influenced the results of the ROC‐based diagnostic performance analyses. The lack of a healthy control group or an FECV group with concurrent diarrhoea and lack of serum biochemistry profiling and histopathological examinations of organ and vascular damage are the other limitations. For this reason, further investigation of the aforementioned biomarkers in a controlled experimental study is recommended in which the severity of all clinical findings is also evaluated histopathologically.

In summary, epithelial surface and intestinal injury repair and pyogranulomatous vasculitis biomarkers were evaluated comparatively in cats with effusive FIP and asymptomatic cats with FECV. The cats with effusive FIP had higher serum IAP, TFF‐3 and I‐FABP concentrations than those with FECV. The cats with thoracic effusion had higher IAP and TFF‐3 concentrations and the cats with abdominal effusion had higher MPO‐ANCA concentrations. Despite the disease being caused by an excessive immune reaction, immunosuppressed cats are known to be predisposed to FIP, suggesting that mounting a successful immune response is a precarious balancing act which has not yet been sufficiently characterized (Malbon et al., 2020). For this reason, in accordance with the promising present results, future studies on suppressing the expression of proteins such as IAP and TFF‐3, which are involved in immunostimulation (Lallès, 2014), and stimulating the expression of proteins such as I‐FABP may shed light on preventing the progression of FECV and predicting the prognosis in cats with effusive FIP. The results of this study also suggest that future studies are required to investigate whether some asymptomatic cats with FECV may benefit from antiviral drugs such as GS‐441524 depending on their intestinal injury and/or repair biomarker concentrations (Addie et al., 2003).

## 5 CONCLUSIONS

According to the higher serum I‐FABP, IAP and TFF‐3 concentrations of the cats with effusive FIP, it was concluded that more severe intestinal and epithelial surface injury develop in cats with effusive FIP. When compared with cats with abdominal effusions, responses such as apoptosis inhibition and immunostimulation to the injured epithelium were more potent in cats with thoracic effusion. This raises the possibility of a more negative outcome in this population. Moreover, one can speculate whether asymptomatic FECV‐infected cats need treatment based on their intestinal injury/repair biomarker concentrations. Further studies are required to investigate whether the suppression of proteins such as IAP and TFF‐3 and stimulation proteins such as I‐FABP could affect the clinical progression of FECV and FIP‐infected cats.

## AUTHOR CONTRIBUTIONS

*Conceptualization; data curation; formal analysis; investigation; methodology; project administration; writing – original draft; writing – review and editing*: Erdem Gülersoy. *Conceptualization; data curation; supervision; writing – original draft; writing – review and editing*: Mahmut Ok. *Data curation; formal analysis; resources; software; writing – original draft; writing – review and editing*: Kamil Üney. *Investigation; writing – original draft; writing – review and editing*: Murat Kaan Durgut. *Data curation; formal analysis; investigation; writing – original draft; writing – review and editing*: Tuğba Melike Parlak. *Investigation; writing – original draft; writing – review and editing*: Yusuf Emre Ekici.

## CONFLICT OF INTEREST STATEMENT

The authors declare no conflicts of interest.

## INSTITUTIONAL REVIEW BOARD STATEMENT

The study protocol was approved by the Faculty of Veterinary Medicine, Selçuk University Local Ethics Committee (Approval number: 2020/43).

### PEER REVIEW

The peer review history for this article is available at https://www.webofscience.com/api/gateway/wos/peer‐review/10.1002/vms3.1299.

## Supporting information



Supporting Information

## Data Availability

Data openly available in a public repository that issues datasets with DOIs.
