# Supplementary material for: Intestinal injury and vasculitis biomarkers in cats with feline enteric coronavirus and effusive feline infectious peritonitis
Source: Vet Med Sci. 2023 Oct 24;9(6):2420–9. doi: 10.1002/vms3.1299 (PMC10650239; doi:10.1002/vms3.1299)
Supplement: Supplementary file 1 — Supporting Information [file VMS3-9-2420-s001.docx]

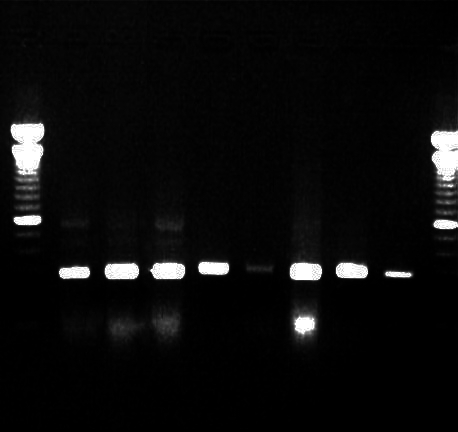


**Supplementary material.** Amplification of FCoV mRNA by RT-PCR. Applied Biosystems Veriti 96-Well thermal cycler (Thermo Fisher Scientific Inc., USA) was used for mRNA amplification and the amplified fragments were visualized in a UV transilluminator and then documented (BTS-20M, Japan).

223 bp product size

C_T_ levels were lower than 29 cycles

Lane 1: 100 Marker

Lane 10: 100 Marker

Lane 2, 3, 7: FECV

Lane 4, 5, 6, 8, 9: FIPV

Primer: P205

Sequence: GGCAACCCGATGTTTAAAACTGG

Orientation: Sense

Target: 3′-UTR

Product size: 223 bp
